# Colonization of Naive Roots from *Populus tremula* × *alba* Involves Successive Waves of Fungi and Bacteria with Different Trophic Abilities

**DOI:** 10.1128/AEM.02541-20

**Published:** 2021-02-26

**Authors:** F. Fracchia, L. Mangeot-Peter, L. Jacquot, F. Martin, C. Veneault-Fourrey, A. Deveau

**Affiliations:** a Université de Lorraine, INRAE, IAM, Nancy, France; b Beijing Advanced Innovation Center for Tree Breeding by Molecular Design, Beijing Forestry University, Haidian District, Beijing, China; Nanjing Agricultural University

**Keywords:** root colonization, microorganisms, metabarcoding, microscopy, poplar

## Abstract

Through their roots, trees interact with a highly complex community of microorganisms belonging to various trophic guilds and contributing to tree nutrition, development, and protection against stresses. Tree roots select for specific microbial species from the bulk soil communities. The root microbiome formation is a dynamic process, but little is known on how the different microorganisms colonize the roots and how the selection occurs. To decipher whether the final composition of the root microbiome is the product of several waves of colonization by different guilds of microorganisms, we planted sterile rooted cuttings of gray poplar obtained from plantlets propagated in axenic conditions in natural poplar stand soil. We analyzed the root microbiome at different time points between 2 and 50 days of culture by combining high-throughput Illumina MiSeq sequencing of the fungal ribosomal DNA internal transcribed spacer and bacterial 16S rRNA amplicons with confocal laser scanning microscopy observations. The microbial colonization of poplar roots took place in three stages, but bacteria and fungi had different dynamics. Root bacterial communities were clearly different from those in the soil after 2 days of culture. In contrast, if fungi were also already colonizing roots after 2 days, the initial communities were very close to that in the soil and were dominated by saprotrophs. They were slowly replaced by endophytes and ectomycorhizal fungi. The replacement of the most abundant fungal and bacterial community members observed in poplar roots over time suggest potential competition effect between microorganisms and/or a selection by the host.

**IMPORTANCE** The tree root microbiome is composed of a very diverse set of bacterial and fungal communities. These microorganisms have a profound impact on tree growth, development, and protection against different types of stress. They mainly originate from the bulk soil and colonize the root system, which provides a unique nutrient-rich environment for a diverse assemblage of microbial communities. In order to better understand how the tree root microbiome is shaped over time, we observed the composition of root-associated microbial communities of naive plantlets of poplar transferred in natural soil. The composition of the final root microbiome relies on a series of colonization stages characterized by the dominance of different fungal guilds and bacterial community members over time. Our observations suggest an early stabilization of bacterial communities, whereas fungal communities are established following a more gradual pattern.

## Abstract (precis)

The tree root microbiome is composed of a very diverse set of bacterial and fungal communities. These microorganisms have a profound impact on tree growth, development, and protection against different types of stress.

## INTRODUCTION

Trees have been recognized as metaorganisms possessing specific microbiomes that are key determinants of tree health and productivity (1). The tree root microbiome, notably its fungi and bacteria, is particularly important since it participates in nutrient and water acquisition and in the protection of trees against pathogens (1, 2). Despite the overall positive effects of microbiome on their hosts, the different members of the microbiome can have contrasting effects: some, such as mycorrhizal symbionts, can be beneficial by promoting plant nutrition and resistance against stresses, while others, such as pathogens, are detrimental (3–5). In addition, species of beneficial microorganisms may differ in their activities, although redundancy between species exist (6). For instance, some bacterial species promote the growth of their hosts by producing phytohormones that stimulate the growth of the root systems, while others facilitate access to key nutrients (7). Similarly, ectomycorrhizal fungi (EcM) that provide nitrogen, phosphorus, and oligonutrients in exchange for carbon can strongly differ among species and strains in their abilities to access key nutrients in soil (8). Though the main abiotic (e.g., edaphic properties and climate) and biotic (e.g., genotype and root exudates) factors that influence the composition of the root microbiome are now well documented (9, 10), little is known on how the assembly of the tree root microbiome occurs.

Roots are mainly colonized by microorganisms found in the surrounding soil that participate as a seed bank. Colonization occurs in a two-step process in which root exudates initiate the recruitment in the rhizosphere, followed by the entry inside the root tissues and a fine-tuning of the communities of the rhizoplane—the root surface—by plant-microbe interactions (9, 11). Root exudates chemistry and dynamic, together with microbial preferences for substrates, determine the assembly of the bacterial community of the rhizosphere in some annual plants (12, 13). However, by comparison, the tree root microbiome is much more complex since it harbors, on top of highly diverse bacterial communities, a plethora of microorganisms with potentially different functional capacities compared to herbaceous plants. Indeed, tree roots are colonized not only by EcM in temperate and boreal forest ecosystems (1) but also by endophytes and saprotrophic fungi, even though their role still remains elusive (14, 15). In addition, some trees of temperate climates, such as maple and alder trees, associate with arbuscular mycorrhizal fungi (AM). Finally, a few trees, such as poplars and eucalypts, are colonized by both EcM and AM. While some of these microorganisms can react to root exudates, others rely on specific molecular dialogues with their hosts to establish themselves in the root system (16). In addition, bacteria and fungi colonizing tree roots may interact together through facilitation (17, 18) and/or competition events (19). Finally, trees are long-lived woody perennial plants with a different management of the nutrient allocation compared to herbaceous and annual plant species such Arabidopsis thaliana or crops (20, 21).

The establishment of the root microbiome is a dynamic process wherein specific microbial communities progressively colonize root systems under both the plant selection and the interactions among microorganisms. Previous works have been done on the total microbial community dynamics in the roots of annual plants or crops (12, 22, 23). Other works on root colonization have been carried out on trees to elucidate the mechanisms of the establishment of the tree root microbiome and of tree root selection. For instance, aspen root colonization by the plant growth-promoting bacteria *Pseudomonas* indicated that the spatial and temporal patterns of colonization of roots was different among the four strains of bacteria and was correlated with the ability of bacteria to form biofilm (24). In pine roots, comparison of the dynamic of root colonization of two EcM fungi revealed different strategies. The ability of *Rhizopogon* to colonize roots rapidly from spores and its important early abundance constrasted with later root colonization and the slow increase in abundance of *Tomentella* (25). Previous work (26, 27) on EcM and AM colonization dynamic in eucalyptus roots showed a successionnal replacement of AM by EcM fungi. Similarly, Lodge (28) showed negative associations among EcM and AM fungi leading to a depletion of AM and an increase in EcM in lateral roots of poplar. Nevertheless, these studies focused on one or few bacterial or fungal species using microbial inoculation and did not look at the overall growth dynamic of the microbiome, including endophytes and saprophytes. However, pioneering studies on ectomycorrhizal and bacterial communities of the roots of pines indicate that the full microbiome is likely subjected to a complex dynamic during the colonization process (29). Investigating the temporal succession of microbial communities colonizing root system of young naive trees is needed in order to help clarify the complex interactions occurring between microbiota and their host trees. *Populus* is a good model to address this question because it is now a well-established model for studying the tree microbiome (15, 30–33) and it hosts both EcM and AM, fungal endophytes, and bacterial communities (34, 35). In addition, poplar clones can be cultivated *in vitro* in sterile conditions, thus limiting genetic variability and allowing to focus solely on the colonization by communities coming from the soil and not vertically transmitted. Last but not least, poplar is an important species in Northern Hemisphere forestry with 80 million hectares of trees in the world (80). In France, poplar culture represent 23% of the annual broadleaf tree yields, and French industries should have difficulties in supply in 2023 (81).

Based on these previous studies, we hypothesized that fungal and bacterial communities originating from the natural soil successively colonized host roots with a progressive replacement of the root microbiota members. To test this hypothesis, we used the gray poplar, *Populus tremula × alba*, as a woody and perennial model organism, and we assessed the dynamics of tree root colonization by fungal and bacterial communities during the first 50 days of contact (T0 to T50) between naive tree roots and soil microbial communities by 16S and internal transcribed sequence (ITS) rRNA gene-targeted Illumina MiSeq sequencing and confocal laser scanning microscopy (CLSM).

## RESULTS

### Plantlet development and ectomycorrhiza formation in natural soil.

**FIG 1:**
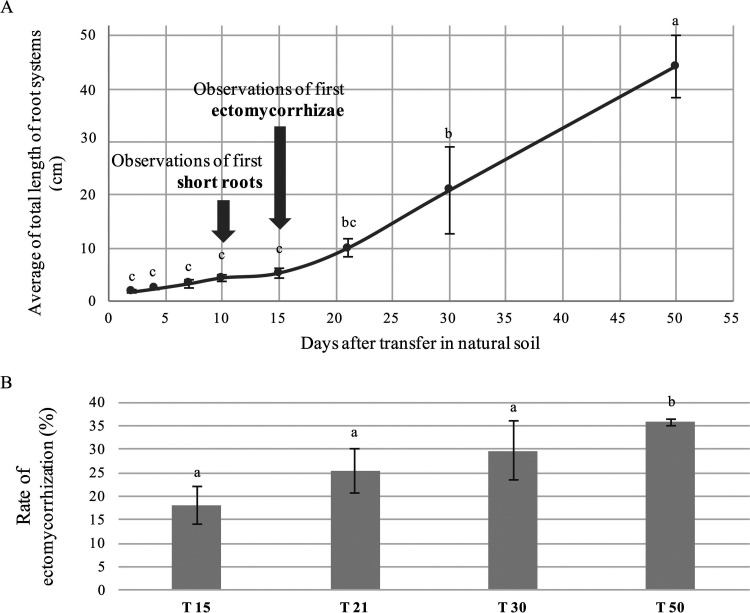
Root development and ectomycorrhiza formation over time. (A) Total length of root system measured at each sampling time from T2 to T50. (B) Ectomycorrhization rate of *Populus* roots from T15 to T50, calculated as the number of fungal colonized lateral roots/total number of lateral roots. Each given value is the average value of seven replicates ± the SE. Different letters denote significant differences between each sampling time (one-way ANOVA, factor-sampling time, *P* < 0.05).

In order to investigate the temporal colonization dynamic of *Populus* roots by fungal and bacterial communities, 3-week-old axenic cuttings of poplar were planted in pots containing natural poplar stand soil (see Fig. S1 in the supplemental material). Monitoring of the root systems growth indicated a slow development of the roots during the first 15 days, followed by an increased growth in the next few weeks (Fig. 1A). First short roots and ectomycorrhizae were observed at 10 (T10) and 15 (T15) days, respectively. The rate of ectomycorrhiza formation regularly increased to reach 37% at 50 days (T50) postplantation and nearly doubled between T15 and T50 (Fig. 1B).

Fungal and bacterial colonization of the roots were tracked using two complementary methods: 16S and ITS rRNA gene-targeted Illumina MiSeq sequencing and confocal microscopy. No amplification of ITS and 16S ribosomal DNA (rDNA) genes was obtained from samples of roots collected before vitroplants were transferred into natural soil. These results are in accordance with CLSM observations concerning fungal colonization since no fungal structure could be visualized at T0 (see Fig. S2). These results validate the axenic status of the *in vitro* root systems.

### Microbial sequencing.

Sequencing of ITS2 and 16S rDNA amplicons was performed on bulk soil and roots DNA samples between T2 and T50. After quality filtering and chimera removal, a total of 909,764 fungal reads and 1,678,387 bacterial reads were kept for further analyses. After taxonomic assignment, elimination of contaminants and completion of normalization by rarefaction, 373 fungal operational taxonomic units (OTUs; 70 ± 1 per sample) and 887 bacterial OTUs (518 ± 6 per sample) were detected. One of the five biological replicates of T4, T10, and T15 ITS and one of T2 and T50 16S data were eliminated from the data after completion of rarefactions.

### Soil microbiome composition.

Since bulk soil is the only reservoir of microorganisms for the colonization of the root system in our conditions, we first analyzed its composition at T0. It was heavily colonized by complex fungal and bacterial communities, as expected from a previous study on soil from the same poplar plantation (36). Twenty bacterial phyla, 111 families, and 633 OTUs were detected. The community was dominated by three phyla: *Acidobacteriota* (27.8 ± 0.2% of the relative abundance), *Verrucomicrobiota* (26.8 ± 1.5%), and *Proteobacteria* (23.1 ± 0.4%; see Table S1). OTUs from genus “*Candidatus* Udeobacter” accounted for 22.0 ± 1.4% of the relative abundance by themselves (see Table S1).

Regarding fungi, Basidiomycota represented the most abundant phylum (45.3 ± 3.1%), followed by Ascomycota (31.0 ± 3.2%) and Mucoromycota (11.7 ± 0.9%; see Table S2). At a lower taxonomic scale, 58 fungal genera, and 156 OTUs were detected in the bulk soil. *Sebacina* was the most dominant and represented up to 17 ± 0.5% of the relative abundance, followed by *Umbelopsis* (10.7 ± 0.8%) and *Mortierella* (9.9 ± 0.5%; see Table S2). Twenty EcM (34.2 ± 3.1% of the relative abundance), 31 saprotrophs (20.3 ± 2.5%), and 6 potential endophytes (9.0 ± 1.1%; see Table S3) were found.

### Overall dynamic of microbial colonization of the root system.

First bacterial and fungal colonizations of *Populus* roots were observed after 2 days of growth (T2). The number of bacterial and fungal OTUs detected in roots was 1.3- and 2.1-fold lower at T2 than in the bulk soil (Table 1, Kruskal-Wallis, adjusted *P* value [P.adj] < 0.05). Then, the diversity of bacterial and fungal communities colonizing the roots evolved differently. Bacterial richness and diversity measured by the Shannon index significantly increased over time to stabilize by ∼21 days. In contrast, fungal richness reached a maximum at 15 days and then decreased, whereas fungal diversity slowly decreased from T15 to T50 (Table 1).

**TABLE 1:** Diversity of bacterial and fungal communities detected in soil and in roots across time^*a*^

Sampling time	Avg ± SE			
	Bacterial richness	Bacterial Shannon index	Fungal richness	Fungal Shannon index
Bulk soil	620.3 ± 6.3*	5.30 ± 0.05*	155.0 ± 1.0*	3.7 ± 0.1*
T2	463.0 ± 31.3 C	3.2 ± 0.3 A	66.0 ± 7.8 ABCD	2.5 ± 0.1
T4	414.8 ± 54.0 C	3.5 ± 0.2 AB	63.3 ± 7.5 BCD	2.4 ± 0.2
T7	494.0 ± 35.1 BC	3.7 ± 0.2 ABC	74.4 ± 8.3 ABC	2.7 ± 0.3
T10	437.4 ± 35.8 C	3.3 ± 0.3 ABC	68.8 ± 11.7 BCD	2.3 ± 0.2
T15	464.0 ± 48.0 C	4.0 ± 0.2 BC	89.8 ± 4.6 A	2.4 ± 0.3
T21	616.8 ± 18.6 A	3.9 ± 0.4 BC	87.6 ± 5.3 AB	2.3 ± 0.1
T30	598.0 ± 46.7 AB	4.4 ± 0.2 C	58.8 ± 3.5 CD	1.7 ± 0.1
T50	648.6 ± 11.0 A	4.8 ± 0.1 C	50.8 ± 3.8 D	1.5 ± 0.2

a Richness and Shannon indexes were calculated for bulk soil and root samples collected at the different sampling times from T2 to T50. Each given value is the average value of four or five replicates. Asterisks (*) denote significant differences between bulk soil and roots collected at T2, and different letters denote significant differences between each sampling time from T2 to T50 (Kruskal-Wallis, correction Bonferroni, Fisher LSD *post hoc* test, P.adj < 0.05).

**FIG 2:**
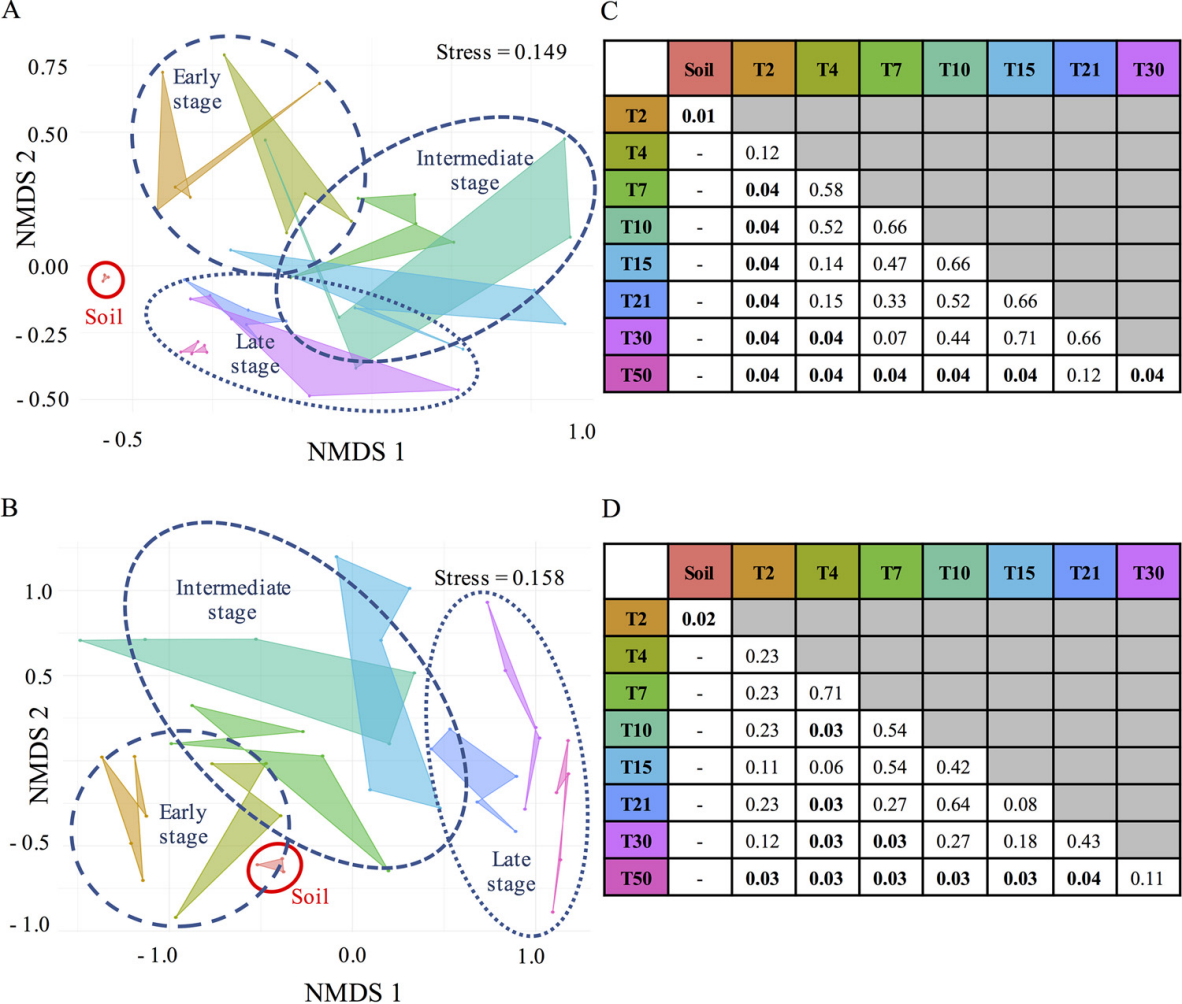
Structure of bacterial and fungal communities colonizing *Populus* roots across time. (A and B) NMDS ordinations of bacterial OTUs (A) and fungal genera (B) across compartments (bulk soil and roots) and sampling times (from T2 to T50) based on Jaccard distance. (C and D) Adjusted *P* values of variances explained based on pairwise comparisons using PERMANOVA on the binary distance for bacterial OTUs (C) and fungal genera (D).

These dynamic changes in richness and diversity were associated with modifications of the structure of the microbial communities compared to the bulk soil, suggesting an early selection of root microbial communities (Fig. 2, Wilcoxon rank sum test, P.adj < 0.05). The structures of root bacterial and fungal communities then progressively evolved from T2 to T50 over time, explaining 45% of the variance for bacteria and 38% for fungi, although the close time points were not statistically different (e.g., T2-T4, T15-T21…, pairwise permutational multivariate analysis of variance [PERMANOVA], P.adj < 0.05; Fig. 2). Three stages of bacterial and fungal colonization were defined based on NMDS (nonmetric multidimensional scaling) graphic representations and pairwise PERMANOVA: an “early” stage from T2 to T4, an “intermediate” stage from T7 to T15, and a “late” stage from T21 to T50.

### Assembly of fungal and bacterial communities in the roots at the early stage.

**FIG 3:**
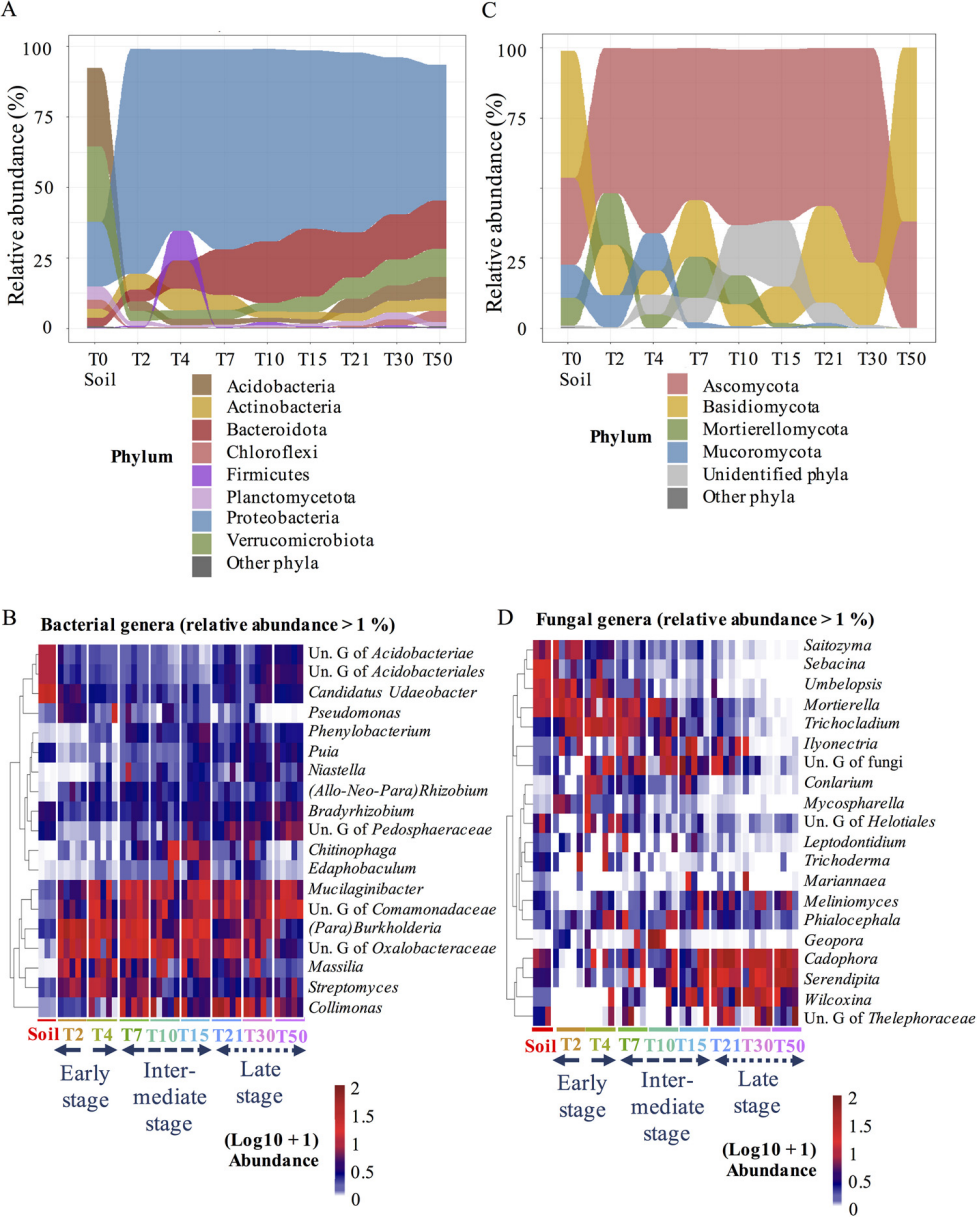
Composition of bacterial and fungal communities colonizing *Populus* roots across time. (A and C) Average representations of the distribution of the most abundant bacterial (A) and fungal (C) phyla (>2% in relative abundance) detected in bulk soil and in *Populus* roots collected at each sampling time from T2 to T50. (B and D) Heatmaps showing the relative abundance of bacterial (B) and fungal (>1% in relative abundance) (D) genera across compartments (bulk soil and roots) and sampling times (from T2 to T50). The cladogram on the left shows the similarity of the microbial taxa in terms of their relative abundance based on Ward’s minimum variance hierarchical clustering. Relative abundances were normalized through log_10_ +1 transformation.

The compositions of both bacterial and fungal communities already strongly differed from that of the bulk soil at T2, although this effect was much more pronounced for bacterial communities (Fig. 3).

Fifteen of the twenty bacterial phyla detected in the bulk soil showed significant differences in relative abundance in roots at T2 (see Table S1, Kruskal-Wallis, P.adj < 0.05). Most striking was the massive colonization of the roots by *Proteobacteria*, which accounted for 79% of all phyla, whereas they were 3.5-fold less abundant in bulk soil (see Table S1, P.adj = 0.034). In contrast, *Acidobacteria* and *Verrucomicrobia* were poorly represented in roots at T2, while they dominated in the bulk soil (see Table S1). Of the 99 genera detected in the roots at T2, the bacterial communities were dominated by 7 genera that all together accounted for 56% of the relative abundance. Among those, four were members of the *Burkholderiaceae* family, and the genus *Burkholderia* accounted by itself for 35 ± 5% of the relative abundance. Members of *Mucilagenobacter* (*Sphingobacteria*), *Pseudomonas* (*Gammaproteobacteria*), and *Streptomyces* (*Actinobacteria*) were also quite abundant, since their relative abundance exceeded 3% at this early time point.

Fungal colonization of the roots was characterized by a significant expansion of Sordariomycetes (5.5×) at the expense of Agaricomycetes (–4.5×), Letiomycetes (−3.5×), and Pezizomycetes (−3×) compared to bulk soil samples (see Table S2, P.adj < 0.01). However, changes in the fungal community composition of the roots were not significant at other taxon levels. As for bacteria, fungal communities detected in roots were dominated by a few genera: nine fungal genera accounted for 72 ± 3.4% (e.g., *Mortierella*, *Umbelopsis*, and *Trichocladium*; see Table S2). However, the relative abundances of these dominant genera were similar in the bulk soil and in roots at T2 to T4, in contrast to bacteria. All dominant genera at this stage except two (*Cortinarius* and *Sebacina*) were saprophytes or putative endophytes. As a consequence, fungal communities of roots were depleted in EcM (−5×) and endophytes (−3.5×) compared to the bulk soil at the early stage (see Table S3, P.adj < 0.01). In particular, *Thelephoraceae* were absent from the root systems, although *Sebacina* spp. were found in low abundance compared to the bulk soil (2.1 ± 1.0% [–8×]).

### Evolution of the compositions of bacterial and fungal communities associated with *Populus* roots over time.

The composition of root bacterial and fungal communities clearly evolved over time, from T2 to T50. The relative abundances of the eight most abundant bacterial phyla (>1%) significantly varied from T2 to T50 (Fig. 3A, see also Table S1; Kruskal-Wallis, P.adj < 0.05). Although still being the most dominant phylum, the proportion of *Proteobacteria* slowly decreased over time from 79.6% (± 2.8) at T2 to 48.6% (± 2.1) at T50 (Fig. 3A, see also Table S1; P.adj <0.05). Proteobacteria were replaced by members of the *Bacteroidota*, *Verrucomicrobiota*, and *Acidobacteriota* and, in a lesser extent, by *Chloroflexi* and *Myxococota* (Fig. 3A). All the genera that dominated in the roots in the early stage displayed decreased relative abundances over time, and all were in the minority at T50. The only exception was the genus *Mucilaginibacter*, which remained among the dominant genera in roots over time. In contrast, the relative abundance of members of the *Chitinophagaceae*, in particular OTUs belonging to the genera *Chitinophaga*, *Edaphobaculum*, and *Sphingomonas*, increased to reach a maximum of 12.9 ± 1.8% at the intermediate stage and then decreased to 5.5 ± 1.8%. Finally, the relative abundance of the *Comamonadaceae*, *Ktedonobacteraceae*, and *Pedosphaeraceae* families and the *Bradyrhizobium* and *Puia* genera significantly increased over time to reach a maximum at T50 (Fig. 3B; see also Table S1; Kruskal-Wallis, *P* < 0.05).

**FIG 4:**
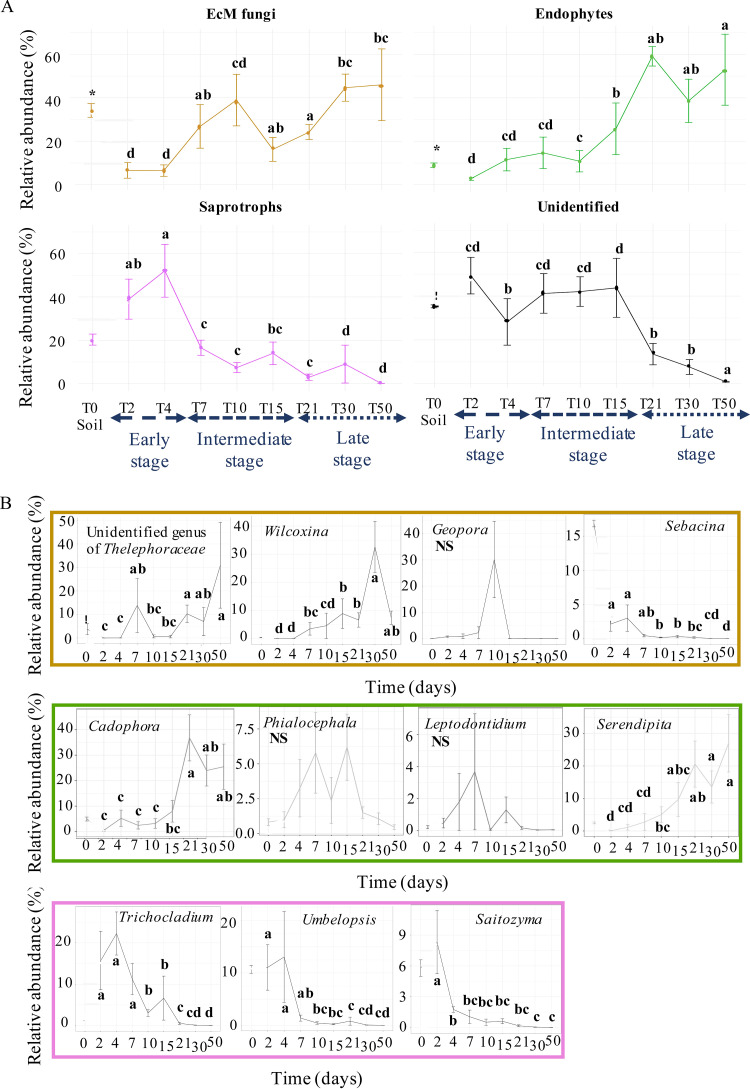
Evolution of fungal guilds in *Populus* roots over time. (A) Relative abundance of the main fungal guilds detected in bulk soil and in *Populus* roots collected from T2 to T50 (3, 4, or 5 replicates ± the SE). (B) Relative abundance of the most abundant EcM (in yellow), saprotrophic fungi (in pink), and fungal endophytes (in green). The asterisks denote significant differences between bulk soil and *Populus* roots collected at T2, and different letters denote significant differences between each sampling time (Kruskal-Wallis, Benjamini-Hochberg correction, Fisher LSD *post hoc* test, P.adj < 0.05).

Like bacteria, the composition of fungal communities in the roots deeply evolved from T2 to T50, although the dynamics slightly differed. The roots were mainly colonized by saprophytic fungi that dominated the soil assemblage until the end of the early stage, suggesting a later selection of fungal communities colonizing *Populus* roots than for bacterial communities. In addition, the fungal colonization was highly variable from one root system to another, particularly at the early time points. Nevertheless, as for bacteria, all dominant taxa at the early stage were fully replaced in the roots by other taxa over time. The relative abundance of Mucoromycota in roots decreased after T7, whereas Mortierellomycota remained abundant until T10 then almost disappeared (Fig. 3C, see also Table S2; Kruskal-Wallis, P.adj < 0.05). Meanwhile, the relative abundance of Basidiomycota slowly increased to become dominant at T50 (61.9 ± 9.2%). This replacement between taxa was accompanied by a change in the ratio between saprophytes, EcM and endophytes in roots: saprotrophs, which represented 40 to 50% of the fungal genera at the early stage (29 genera, e.g., *Trichocladium*, *Saitozyma*, and *Umbelopsis*; Fig. 3D), were rapidly replaced by EcM at the intermediate stage (18 genera), while the proportion of endophytes continuously increased to reach 59 ± 5% in the late stage (four genera, Fig. 4A). Interestingly, successional replacements occurred within EcM and endophyte guilds. The *Sebacina* and *Geopora* EcM were detected in some roots systems at the early and intermediate type points, respectively, but none of them persisted over time, whereas *Thelephoraceae* and *Wilcoxinia* gradually took over the root systems. Similarly, the endophytes *Leptodontidium* and *Phialocephala* were found only at the intermediate stage, whereas *Serendipita* and *Cadophora*, which had already been detected in low abundance at T2, significantly increased from the intermediate to the late stage, representing, respectively, 27.0 ± 8.8% and 25.5 ± 8.9% of the relative abundance at T50 (Fig. 4B), respectively, making them the major trophic group at T50 (52.5 ± 16.3%; see Table S3).

**FIG 5:**
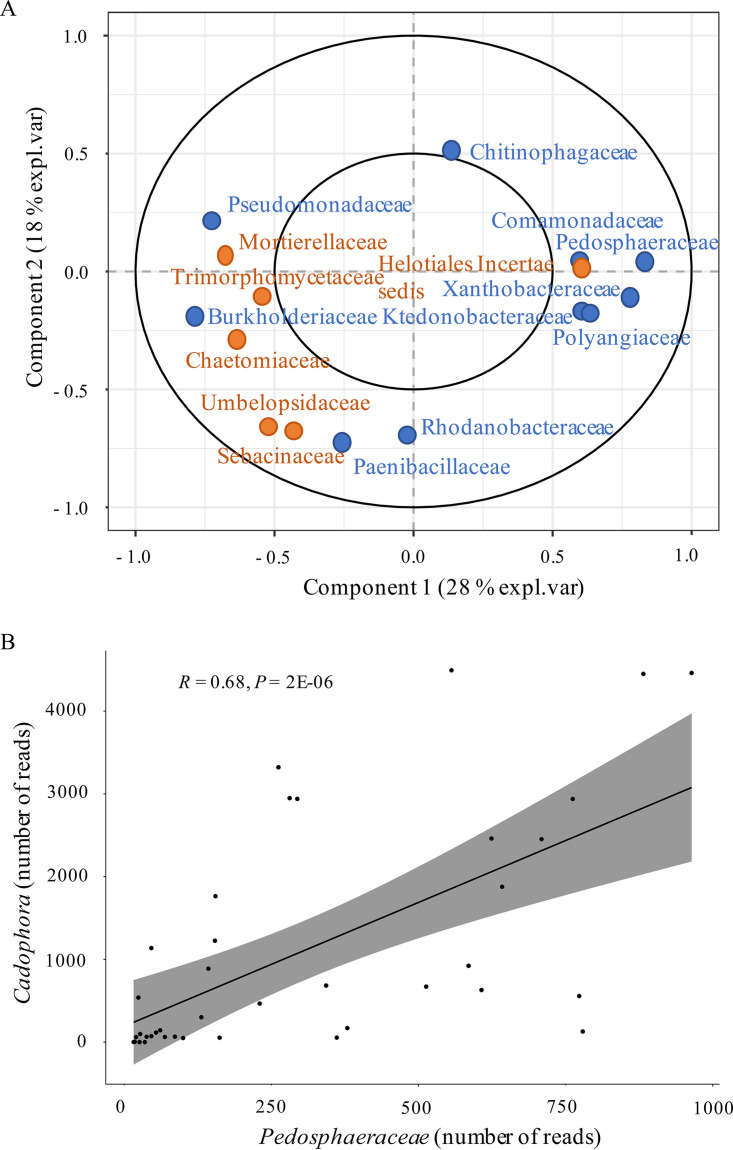
Association patterns between the most dominant microbial communities detected in roots over time. (A) Sparse sPLS of 18 bacterial (in blue) and 13 fungal (in orange) dominant families detected in roots over time. (B) Correlation between the number of reads of *Cadophora* endophyte fungi and the number of reads of an unknown genus from the *Pedosphaeraceae* family (Spearman correlation test, *P* < 0.01).

It is likely that some fungi and bacteria interact during the colonization of roots. To uncover such potential interactions, we looked for association patterns between the 13 fungal and 18 bacterial dominant families by sparse partial least square regression (sPLS). Strong associations were found between the relative abundances of *Burkholderiaceae* and *Chaetomiaceae* and between *Pedosphaeraceae* and *Heliotiales* incertae sedis, and both were linearly correlated (Spearman *P* < 0.01, Fig. 5). In particular, the presence of unknown genera from the *Pedosphaeraceae* family (*Verrucomicrobia*) correlated with that of the endophytic fungus *Cadophora* (*r*^2^ = 0.68, *P* = 2E−06).

### Monitoring of fungal colonization in *Populus* roots by CLSM.

The MiSeq results provided global information about the structure and composition of microbial communities without knowledge about their spatial distribution and their physical interaction with the roots and with other microorganisms. In order to deepen our understanding of the process of root colonization and its dynamic, fungal colonization of the roots was also followed by CLSM.

**FIG 6:**
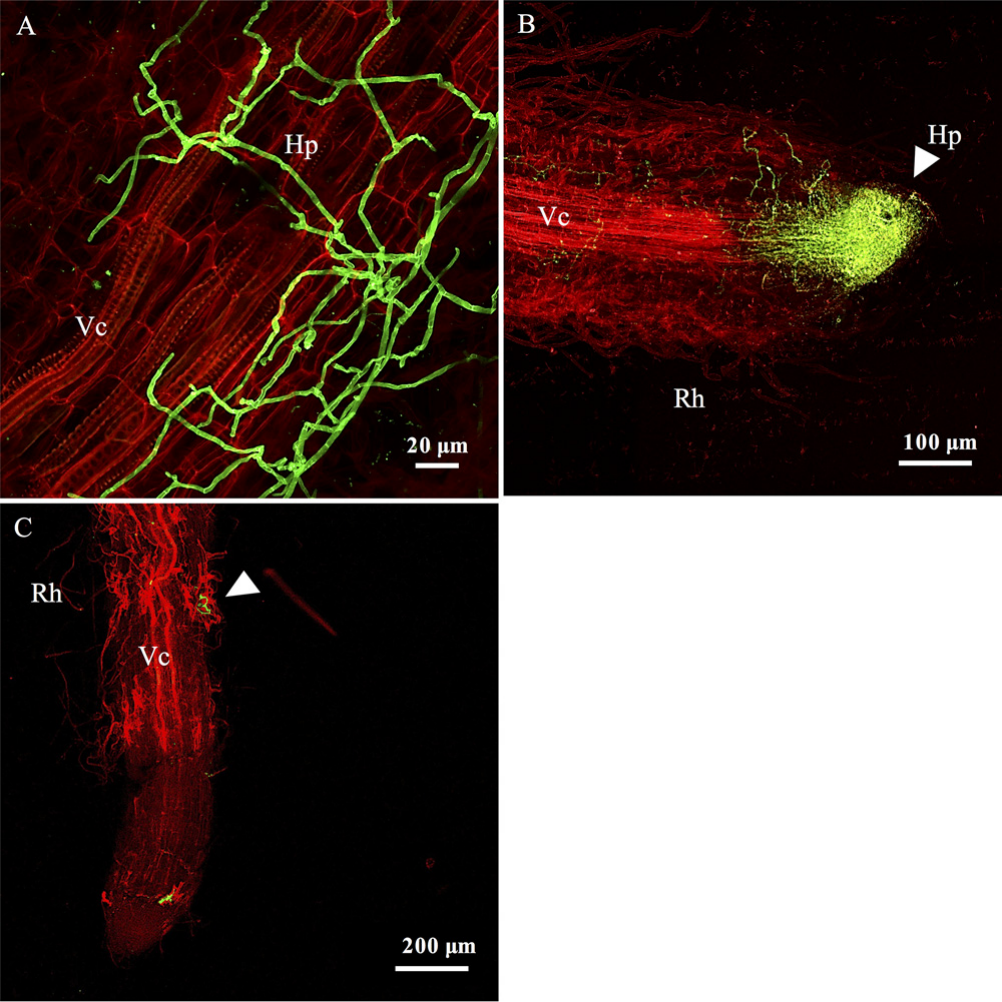
Early stage of the fungal colonization dynamic. Confocal microscopy images of poplar roots colonized by fungi after 4 days of culture. (A) Extracellular hyphae surrounding a root after 4 days of culture. (B) Hypha accumulation at the apex of the root after 4 days of culture. (C) Hyphae on root hairs after 4 days of culture. Fungal structures appear in green after WGA-Alexa Fluor 488 staining, whereas root cell walls appear in red after propidium iodide staining. Ap, apex; Vc, vascular cylinder; Hp, hyphae; Rh, root hair. Arrowheads indicate fungal hypha colonization.

As for high-throughput sequencing, the first fungal presence was detected by CLSM between T2 and T4 (Fig. 6A). We observed spores and hyphae colonizing the surface of root systems mainly from the apex (Fig. 6B). These colonizations were very heterogeneous from one sample to another; some root apices were entirely surrounded by fungal mycelia, while others had few hyphae (Fig. 6C). The fungal hyphae were septate, with a diameter of <1 μm, and we observed a very low diversity of morphologies. After 4 days of growth, we observed by light microscopy the presence of melanized septate hyphae which were not stained by wheat germ agglutinin-Alexa Fluor (WGA) (see Fig. S3). Their hyphae were either extracellular or intercellular, but it was difficult to assess whether they were intracellular or within the apoplastic space.

**FIG 7:**
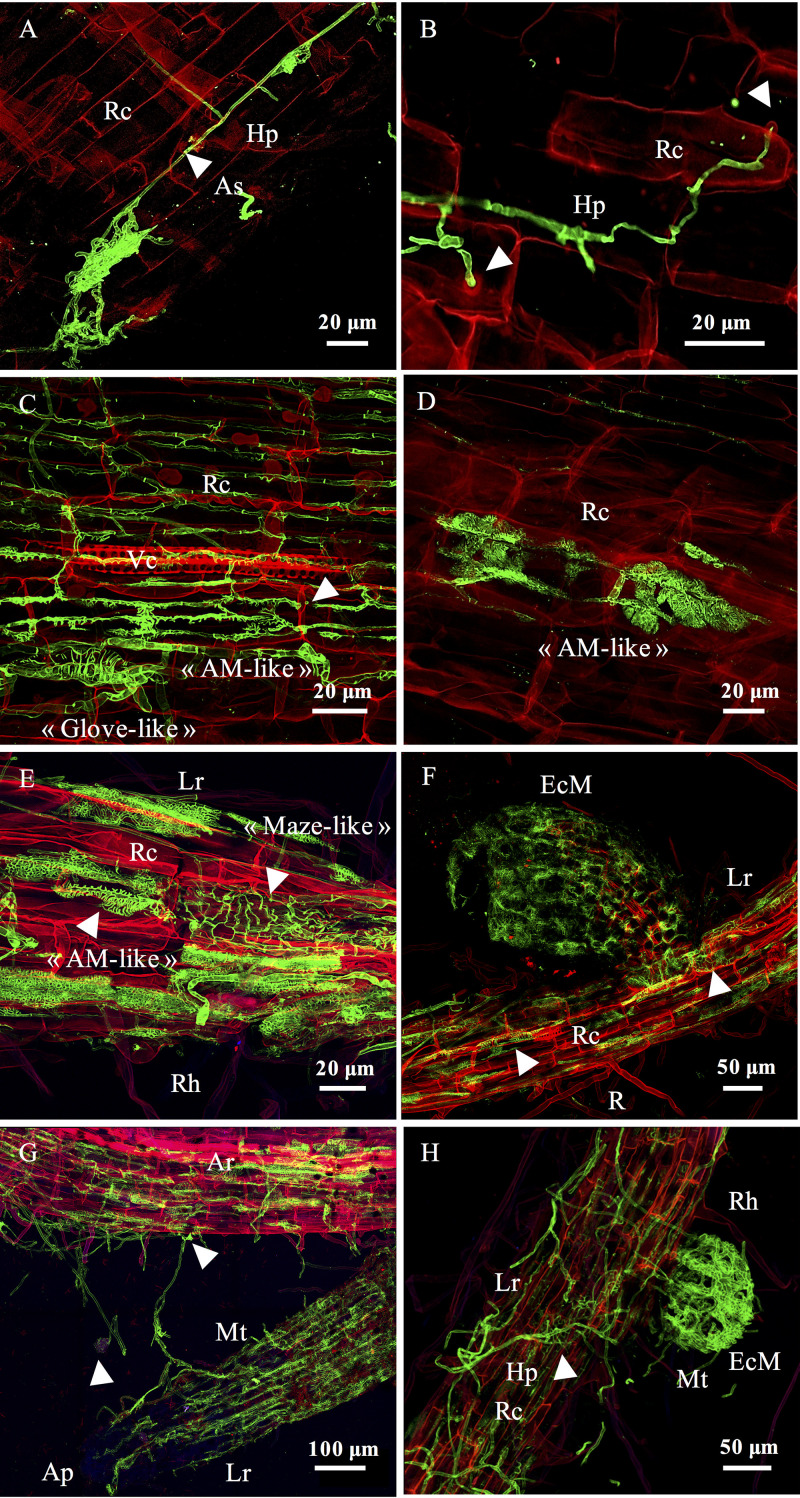
Intermediate and late stages of dynamic fungal colonization. Confocal microscopy images show poplar roots colonized by fungi after 7 to 15 days (intermediate stage) and after 21 to 50 days (late stage) of culture. (A) Development of fungal hyphae in the apoplastic space of cortical poplar cells after 7 days of culture. (B) Intracellular hyphal penetration in root cells after 7 days of culture. Arrowheads indicate the deformation of the root cell under hyphal pressure. (C) Coexisting fungal morphologies (“arbuscular like” and “glove-hand like”) within the same root region after 15 days of culture. Arrowheads indicate hyphal intracellular penetration. (D) “Arbuscule-like” morphology observed in poplar root after 15 days of culture. (E) At least three different morphologies coexist within the same root region after 21 days of culture. The white arrowheads indicate a “maze-like” structure and an “arbuscule-like” structure. (F) Mycorrhizal formation with coexisting fungal morphologies after 30 days of culture. The white arrowheads indicate an “arbuscule-like” structure and the presence of “maze-like” fungal morphology that seemed to be linked to the “glove-hand-like” morphology and the EcM forming structure. (G) Hyphal propagation at T30 between the adventive root to the lateral root, forming a probable EcM. The white arrowheads indicate a germinated spore and a “hand-glove-like” morphology. (H) EcM formation after 30 days of culture. Arrowheads indicate the “maze-like” structure from which EcM seemed to originate. Fungal structures appear in green after WGA-Alexa Fluor 488 staining, while root cell walls appear in red after straining with propidium iodide. Ar, adventive root; Ap, apex; Hp, hyphae; Lr, lateral root; Mt, mantle; Rc, root cell; Rh, root hair.

We detected an increased density of fungal morphologies by CLSM after 7 days. Hyphae developed either between root cells, propagating in the apoplastic compartments particularly around epidermic regions (intercellular), or directly into root cells (intracellular) (Fig. 7A and B).

The apoplastic colonization stayed heterogeneous along the roots and was dominantly present at the apex and in the root elongation zone. After 10 days, we observed an increase of both apoplastic and intracellular colonization. Indeed, hyphae were propagating from cell to cell by going through the root cell walls, and we were even able to see the pressure of the hyphae on the cell walls (Fig. 7B). Even though the global fungal diversity of morphologies remained poor at this stage of development, we noted the presence of septate and nonseptate hyphae with diameters either inferior or superior to 1 μm, and we still observed the presence of melanized hyphae. After 15 days of culture, we observed an important increase in fungal density and morphological diversity in the root systems. We identified within the same root region the occurrence of distinct fungal morphologies with the dominance of two major structures (Fig. 7C). We detected the first dominant morphology in the intracellular compartment propagating from cell to cell and displaying an arbuscular mycorrhiza-like shape (Fig. 7D). The diameter of the hyphae was <1 μm, and hyphae developed by going through the cell walls from the epidermic to the central cells, forming a grid-shaped network. The second dominant morphology seemed to propagate in both intracellular and intercellular compartments, forming a “glove-like” shape with hyphae diameter closer to 5 μm (Fig. 7C). This structure seemed to surround the root cell, reminiscent of the Hartig net structure observed in ectomycorrhiza. The development of lateral roots after 15 days of culture was correlated with the establishment of the first distinct ectomycorrhizal structures (see Fig. S3). Most EcM root tips already exhibited a mantle and a Hartig net (Fig. 7); however, some EcM did not have a fully formed mantle, and hyphal colonization seemed to originate from the adventive root system. The density of colonization and the occurrence of ectomycorrhizal structures were heterogeneous among the different root systems. Nevertheless, many fungal morphologies were present within the same region, on both lateral and adventive roots (see Fig. S4).

From 21 to 50 days of growth, we observed a global increase of the fungal density within the same root region, with some roots systems being colonized from the apex to the top of the root at 50 days, even though it remained heterogeneous between the different root systems. We still observed both inter- or intracellular melanized septate hyphae, and we detected two new abundant fungal morphologies that were sometimes located within the same root region. The first structure was developing in the intracellular compartment in both adventive and lateral roots, displaying a globular shape with a hyphal diameter of <1 μm (Fig. 7E). The second morphology was only present in lateral and mycorrhized roots, with a hyphal diameter greater than 1 μm and displaying a “maze-like” structure (Fig. 7E). Its location between the “inter and intra” compartments, as well as its origin, was difficult to determine, but it is noteworthy that it was often associated with, and seemed to develop within, ectomycorrhizal structures (Fig. 7F). In addition, we observed fungal structures developing between the adventive and the lateral root forming a potential EcM (Fig. 7G). The apex of the lateral root was not colonized by any fungal structure, suggesting that the EcM forming originated from preexisting fungal structures on the adventive root. We also detected the presence of germinating spores with emerging hyphae colonizing the root cells (Fig. 7G). We observed an increase in EcM establishment (Fig. 7H), and we assessed by mycorrhizal counts using CLSM that 37 ± 1% of the lateral roots were forming ectomycorrhizal structures at the end of the experiment, even if their presence was also variable depending on the root systems.

Regarding the lateral roots, we observed the successional replacement of the “arbuscule-like” structures to the benefit of the “glove-like” structures, surrounding the root cells and looking like the Hartig net and EcM. We did not observe this pattern in the adventive roots, where the “arbuscule-like” morphologies continued to develop among the “glove-like” structures.

## DISCUSSION

The establishment of the plant root microbiome is a dynamic process involving rich communities of microorganisms with distinct trophic modes and functional abilites (13). It relies on a complex set of interactions between the roots and microorganisms and between microorganisms themselves. While studies of the root colonization dynamic by bacteria and, to a lesser extent, by fungi have been performed on several herbaceous plants and crops, few studies have been performed thus far to understand the early colonization dynamic of tree roots by complex microbial communities (29). Here, we developed a microcosm experiment to grow axenic poplars in natural soil and to track the colonization of the root system by microorganisms. The transfer of plantlets from axenic conditions to the microcosm did not induce visible stress to plantlets since they grew normally and developped short roots and EcM symbiosis at the same rates and with the same timing as in other systems (37). In addition, we observed a rapid and dynamic colonization of the root system by both fungi and bacteria. We were able to track both AM and EcM fungi, suggesting that our microcosm allowed a normal development and colonization of the root system. We are not aware of any prior study investigating the primary steps of the spatiotemporal colonization of tree roots by fungi and bacteria.

### *Populus* root colonization dynamics differed between fungal and bacterial communities.

Previous studies suggest that the root microbiome assemble from the surrounding soil in a two-step process. Rhizodeposition would first fuel a recruitment near the roots within a few days and is followed by entry inside the roots and regulation of the community composition by the plant-microbe interactions (12, 23, 38). In accordance with this model, colonization of the poplar adventitious roots started within 2 days for both fungi and bacteria, and the initial root microbial community gradually evolved over time. However, the degree of selection and the pattern of evolution differed greatly between bacterial and fungal communities. Root bacterial communities were already clearly different from the bulk soil only after 2 days, suggesting that a very early selection was operating for bacteria. In rice, the first bacterial recruitment occurred by ∼24 h (23). Sampling at earlier time points would be necessary to determine with more precision the exact timing and the very first steps of the bacterial colonization of the poplar roots. The bacterial diversity increased and then stabilized by approximately 15 to 21 days in a dynamic similar to that for rice (23), but the community composition kept evolving until 50 days. Long-term evolution of short lateral root and EcM tip-bacterial communites was observed in pine for 24 weeks (29), indicating that the bacterial community may not have reached the equilibrium by 50 days in the present case. However, no real “climax” can be expected regarding the microbiome of tree roots for more than a few months since root microbial communities evolve with seasons (8, 39) and the age of trees (40).

In contrast to bacteria, the root fungal community partially mirrored the one of the bulk soil at the early stage. It started to clearly diverge from the soil community at the intermediate stage, suggesting a later selection process leading to a reduction of fungal diversity in the roots. This is likely due to (i) the differences in the growth capacities of the three trophic guilds of fungi colonizing the roots and (ii) the molecular dialogue necessary for the establishment of AM, EcM, and endophytes in roots. Indeed, if saprophytes and some endophytes (e.g., *Mortierella* and *Ilyonectria*) can rapidly develop on a carbon-rich source, EcM are generally slow growers, while AM require stimulation by plant strigolactone to develop (41). Endophytic fungi could directly interact with tree roots and promote host growth indirectly by manipulating the microbial community composition and functioning and manipulating host phytohormones (15). Thus, both mycorhizal and endophytic types are less likely to colonize roots within a few days, even though reads of some EcM were already detected in low abundance at the early stage.

Altogether, our results suggest that bacteria and fungi react differently to tree selection factors or that the tree would select the bacterial and the fungal community through different processes. Bacteria and some fungi would be rapidly and strongly responsive to root exudates, while other fungi would be sensitive to more specific signals (e.g., strigolactones, flavonoids, etc.). The composition of poplar root exudates is not known, to the best of our knowledge. We have detected several sugars, such as mannitol, sucrose, glucose, and arabinose, known to attract microorganisms in root exudates of axenic *Populus tremula × alba* after only 8 h in hydroponic solution (data not shown). Future experiments should investigate the composition of poplar root exudates over time in order to differentiate the host tree selection and the effects of competition that may exist between different members of the root microbiome.

### Saprotrophs dominated early microbial communities but were counterselected over time.

*Proteobacteria* and particularly *Burkholderiaceae* dominated the early root bacterial community. This is in accordance with previous studies that showed a significant enrichment of OTUs from *Proteobacteria* and from *Burkholderia* in the roots of different tree species (6, 11, 29, 30, 42). Representative members of the *Burkholderiaceae* family tend to develop on root exudates (12, 43, 44). Similarly, fungal communities detected after 2 and 4 days of growth in natural soil were dominated by saprotrophs. These observations suggest that saprotrophs, rare in soil, are the fastest colonizers of tree roots; this is certainly due to newly available carbon sources from the plant and root exudates (13). Endophytes such as *Mortierella* are also likely able to quickly grow on root exudates. Members of the *Mortierella* genus are commonly detected in the soils of forests and poplar plantations (30, 32, 45). Although their ecological role is poorly understood, these fungi are characterized by their rapid growth when encoutering rich media (46). This dominance of saprotrophs among the microbial communities at early time points advocates for an important role of root exudates and particularly primary metabolites in the early colonization of the roots by microorganisms. In accordance with this hypothesis, fungal colonization was limited to the surfaces of the roots and occurred mainly at the apex (Fig. 6A), the area where most of the primary metabolites are exudated (47).

 Nevertheless, almost all taxa that dominated at the early stage, whether fungal or bacterial, were replaced over time. For instance, the relative abundance of members of the *Burkholderiaceae* family decreased for the benefit of other well-known tree root colonizers such as *Bradyrhizobium*, *Rhizobacter*, or *Sphingomonas* (48). Several phenomena could explain such evolution of the microbial communities: competition between microorganisms, the slow growth of late comers, the evolution of the composition of the root exudates, selection by the tree, and cross-kingdom interactions between bacteria and fungi. Previous work on the annual grass *Avena fatua* showed that the dynamics of root exudate chemistry and the bacterial preferences for substrates drive the bacterial community assembly over the full season of growth (12, 13). Whether such a mechanism also applies in a shorter period of time needs to be tested, since nothing is known thus far regarding the dynamics of poplar root exudates during root development. Secondary metabolites could also play a role in the process. Association studies between the level of salicylates in poplar and microbial community composition suggest that colonization of the rhizosphere by a number of bacterial taxa and fungi (e.g., Mortierellomycota) could be influenced by the levels of salicylic acid, populin, and tremuloidin (33). Whether these compounds participate in the early selection of the microbiome needs to be tested. Finally, one cannot exclude also that the bulk soil community that serves as a reservoir evolved over time, although this is less likely based on our previous experiments (36).

More unexpected is the very early detection reads corresponding to EcM in the roots, before short lateral roots start to develop. Among these, some such as *Sebacina* or *Laccaria* were present already at the early stage but did not further develop. Others were detected at T7 before the development of the short roots and were further able to form true ectomycorrhizae (*Thelephoraceae* and *Wilcoxina*). Similar early colonization of primary root by EcM was found in *in vitro* experiments when inoculating eucalyptus roots with the ectomycorrhizal fungi *Pisolithus tinctorius* and *Paxillus involutus* (49) and Betula pendula with *Paxillus involutus* (50). Indeed, both studies found evidence of hyphal attachment to the roots within 2 days of inoculation with an accumulation of hyphae at the root apices. These observations suggest that EcM can colonize the adventive primary roots before the formation of short lateral roots. Whether this step favors EcM formation and requires a specific molecular dialog between the fungi and the plant for the establishment of the symbiosis should be evaluated in future studies.

### The dominance of fungal saprotrophs versus EcM and endophyte fungi was reversed over time in *Populus* roots.

The relative abundance of saprotrophic fungi decreased after 4 days, in contrast to the relative abundance of EcM and endophytes, which increased during the intermediate and late stages of root colonization, reaching 99% in relative abundance by the end of the experiment according to metabarcoding data (Fig. 4A). CLSM also revealed the abundant presence of AM at the intermediate and late stages, whereas AM reads were almost completely absent from metabarcoding data (see Table S3). Analysis of Illumina MiSeq raw data before rarefaction indicated that reads corresponding to 10 OTUs belonging to the genera *Rhizophagus* and *Funneliformis* and the Archeosporales and Paraglomales orders could be detected in the roots but at very low levels that do not reflect the colonization of roots by AM as observed by CLSM. Such artifacts and difficulties in properly tracking AM fungi in poplar roots by high-throughput sequencing have already been highlighted by several authors (45, 51, 52), who suggest the use of additional methods to analyze AM interactions with poplar roots. Nevertheless, the combination of metabarcoding data and microscopy observations made it possible to draw the sequential events of the colonization of naive poplar roots by fungi from the soil. Upon secretion of exudates in the rhizosphere, saprophytic fungi massively grew at the apices and on the surfaces of the roots for a few days. Whether plant defense mechanisms or interference competition with mycorrhizal fungi and/or endophytes (53) put an end to their development needs to be determined. Meanwhile, endophytes, AM, and EcM slowly developed on the surface and started colonizing the inner tissues between T4 (*Phialocephala*, *Cadophora*, and *Leptodontidium*) and T7 (AM, EcM, and other endophytes [potentially, *Serendipita*]). The detection of dark septate endophytes (DSE) by amplicon sequencing coincided with the observation melanized septate hyphae in the roots, suggesting that those could be DSE despite the absence of microsclerotia. At 10 days, the first short roots formed and were colonized by at least AM, presumed DSE, and EcM. However, if AM continued to be present in the adventive roots, they did not maintain themselves over time in short roots. The replacement of AM by EcM has been previously documented in eucalyptus (26, 27, 54) and poplar (28). However, these studies were performed on a long-time scale, from 5 months to years, looking at the fungal colonization dynamic of already mature trees, and the mechanisms involved in such processes are unknown.

At 30 days, the inner tissues of adventitious roots were massively colonized but by fewer genera than at earlier time points, mainly by the endophytes *Serendipita*, *Ilyonectria*, and *Cadaphora*, whereas EcM from *Thelephoraceae* and *Wilcoxinia* likely developed functional ectomycorrhizae with the Hartig net (Fig. 3D, Fig. 7F to H). As reported previously (55), we noted by microscopy that some presumed DSE were often associated with EcM. In addition to presumed DSE, we also observed within a single ectomycorrhizal structures a diverse range of fungal morphologies, as also described recently in eucalyptus EcM (56). These observations suggest that ectomycorrhizae in natural settings are made of more complex communities than “only” the root, the ectomycorrhizal fungus, and associated bacteria; these communities would also include several additional endophytes whose relationship with the rest of the root-microbe community remains to be deciphered.

Fungal community compositions at T30 and T50 are very similar, suggesting that an equilibrium may have been reached. However, this equilbrium would be only temporary since it is known that the fungal community of trees evolves with seasons (8, 57) and all along their life span (58).

Interestingly, we observed the successional turnover of distinct EcM and endophytic fungi in poplar roots. In both cases, fungal species that started to develop at the early to intermediate stages but did not persist at the late stage are well-known members of the poplar root microbiome (e.g., *Mortierella*, *Umbelopsis*, and *Sebacina*), suggesting that their exclusion is not due to a defensive reaction of the plant (15, 30, 52). The competition abilities of endophytes investigated so far (15) show that a single endophyte species can shift the whole community of root-associated microorganisms. In addition, the competition and priority effects in EcM have been deeply scrutinized in the past (59). Both mechanisms are considered to play an important role in structuring EcM communities. In the present case, EcM genera that dominated in bulk soil (e.g., *Sebacina*) and at an early time point did not take over the root system, suggesting that the priority effect was not the main process involved in the successional events of colonization. However, the important variability in the colonization of root systems by fungi at the early time points may result from stochastic events and priority effects. Since *Thelephora* and *Wilcoxina* are considered to be highly competitive species (60, 61), we hypothesize that competitive exclusion is likely involved in this process. Potential interactions with other members of the microbiome may also be at play.

### Conclusions.

In conclusion, we demonstrated here that the bacterial and fungal communities of bulk soil successively colonized *Populus* roots. This colonization took place in three major stages. The early stage was characterized by a massive colonization of the naive roots by *Proteobacteria* members and saprotrophic fungi, while the intermediate and late stages were characterized by an increase in *Bacteroidota*, *Verrucomicrobiota*, and *Acidobacteriota* (even if *Proteobacteria* still dominated bacterial communities), as well as endophytic fungi and EcM. The establishment of root bacterial communities was stable earlier than fungal communities, suggesting different establishment processes between bacteria and fungi. Our observations constitute a first phase of exploration of the establishment of tree-microbe interactions as soon as roots appear and come into contact with the bulk soil. Future experiments should investigate the mechanisms involved in the formation of the root microbiome to disentangle the relative contributions of root exudates, plant defense, and competition among microorganisms in this process.

## MATERIALS AND METHODS

### Biological material and sample preparation.

*Populus tremula × alba* (INRAE clone 717-1B4) vitroplants were cultivated on Musharige-Skood medium (MS) supplemented with indole-3-butyric acid (IBA) (2 ml liter^−1^) during 1 week before transfering them on MS for 2 weeks at 24°C in a growth chamber (photoperiodicity, 16 h; light intensity, 150 μmol m^−2^ s^−1^) until the root systems were developed, as described previously (62). Soil was collected from an 18-year-old poplar stand planted with *Populus trichocarpa* × *deltoides* and located in Champenoux, France (48°51′46″N, 2°17′15″E). The first soil horizon (0 to 15 cm) was collected over an area of about 1 m^2^ (i.e., ∼50 kg of soil) and after pruning of brambles and adventitious plants and litter removal with a rake. The soil was maintained at room temperature, homogenized through sifting at 2 mm, and fixed at 75% humidity. Portions (50 g) of bulk soil were sampled in triplicate and stored at −20°C until DNA extraction.

Rooted vitroplants were selected to be homogeneous in terms of the size of the aerial part and the root system (i.e., ∼1 cm long for aerial parts and ∼2 cm for roots). Selected vitroplants were transplanted in natural soil in transparent plastic pots with a filtered cover allowing gas exchange and a dark area at the ground level to prevent alga development. Plants were cultivated in a growth chamber (photoperiodicity, 16 h; light intensity, 150 μmol m^−2^ s^−1^). Humidity in pots was maintained at 75% during the experiment by weighing pots and regular watering. Vitroplants were harvested after 0, 2, 4, 7, 10, 15, 21, 30, and 50 days of growth (see Fig. S1). At the beginning of the experiment (time point “T0”) and, at each time point, the root systems of five plants were harvested, rinsed with sterile water in order to remove all soil particles from the rhizosphere, placed next to a ruler, photographed (Nikon Coolpix P530), frozen in liquid nitrogen, and stored at −20°C until DNA extraction. Two additional plants were harvested, and the roots were fixed in a solution containing 1 volume of 1× phosphate-buffered saline (PBS; 0.13 M NaCl, 7 mM Na_2_HPO_4_, 3 mM NaH_2_PO_4_ [pH 7.2]) for 3 volumes of 3% paraformaldehyde (PFA) overnight at 4°C (63). At the T30 and T50 time points, the root system was sufficiently developed to be split into two equal parts to perform two technical approaches on all plants.

### Vitroplant growth and EcM root colonization monitoring.

Total areas of root systems were measured for each vitroplant collected at the different time points on scan images using ImageJ (64) before freezing in liquid nitrogen or PFA fixation. The mycorrhization rate of each vitroplants was quantified as previously described (65). Briefly, each root system was observed under a dissecting microscope. For each root system, 100 short roots were randomly examined and assessed as mycorrhizal or nonmycorrhizal. The mycorrhization rate is defined as the number of mycorrhizal roots observed divided by the total number of short roots examined.

### Confocal laser scanning microscopy.

Staining procedures of root systems and fungi were adapted from (66) protocol. In brief, fixed root systems were washed three times in one volume of 1× PBS and a last wash in 1 volume of PBS/1 volume of 96% ethanol before clearing them during 2 h at 90°C in 20% KOH. After three washes in distilled water, the samples were incubated overnight in 1× PBS containing 10 μg ml^−1^ WGA-Alexa Fluor 488 (Thermo Fisher Scientific, Waltham, MA), a specific marker of the chitin of fungal cell walls. The root systems were then washed in 1× PBS, followed by incubation for 15 min in 1× PBS containing 10 μg ml^−1^ of propidium iodide (a DNA intercaling agent that is excluded by intact cell membranes and stains plant walls regardless of cells viability [67]), followed by another three washes in 1× PBS. Samples were mounted between slide and cover slip with a drop of SlowFade solution (Life Technologies). All root samples were observed with a Zeiss LSM 780 confocal laser scanning microscope (Zeiss International). WGA-AF488 was excited using a 488-nm excitation wavelength and detected at 500 to 540 nm, whereas a 561-nm excitation wavelength and detection at 580 to 660 nm were used with propidium iodide. Maximum intensity projections were performed using ZEN software with a z-stack width of 30 to 50 μm.

### Staining for optic microscopy and observation.

Blue staining of fungal stuctures was adapted from previously published studies (66, 68). Cleared roots were incubated at 90°C in 10% KOH for 20 min. After a few washes in distilled water, root systems were incubated for 10 min in 0.1 N HCl at room temperature. We removed the HCl without washing, and we incubated the root systems for 30 min at 90°C in acidified ink (5% Waterman ink, 20% lactic acid, 75% water). Finally, the roots were washed in distilled water before being mounted between slides and cover slips with a drop of 20% glycerol for observation under the Olympus BX41 optic microscope (Jenoptik Progres Gryphax camera, AxioVision v4.8.2 software). Light microscopy was used to look for melanized fungi that were not stained by WGA-Alexa Fluor 488.

### DNA extraction, Illumina MiSeq amplicon sequencing, and quantification of microorganisms on roots.

Approximately 250 mg of bulk soil samples was used for each individual soil DNA extraction. Soil DNA was extracted using a DNeasy PowerSoil kit according to the protocol provided by the manufacturer (Qiagen, Venlo, Netherlands). The root system of each vitroplant was crushed in liquid nitrogen with a mortar and pestle, and 50 mg of root tissue was used to extract DNA using a DNeasy Powerplant kit (Qiagen). The DNA for all extractions was quantified by using a NanoDrop 1000 spectrophotometer (NanoDrop Products, Wilmington, DE).

A two-step PCR approach was performed to barcode tag templates with frameshifting nucleotide primers, as described previously (11). Forward and reverse primer mixtures were used to maximize the phylogenetic coverage of bacteria and fungi. Primer mixtures for tagging bacterial amplicons were composed of four forward and two reverse 515F and 806R primers screening the 16S rRNA V4 gene region in equal concentrations (0.1 μM; Table 2) (36). Primer mixtures for tagging fungal amplicons were composed of six forward primers and one reverse primer for the ITS2 rRNA region at equal concentrations (0.1 μM; Table 2) (36). To inhibit plant material amplification, a mixture of peptide nucleotide acid (PNA) blockers targeted plant mitochondrial and chloroplast 16S rRNA genes, and plant ITS nuclear rRNA genes were added in PCR mixes (0.75 μl of PNA probe, 5 nM for 2 μl of isolated DNA at ∼10 ng/μl) (36). PCRs were performed for three replicates of each sample (2 μl of isolated DNA at ∼10 ng/μl) using 2.5× Phusion Flash high-fidelity master mix (Thermo Scientific) with 1.5 μl of a forward and reverse primer mix, 0.75 μl of PNA probe (5 nM), and 8.5 μl of 0.2-μm-filtered UV-treated DNA-free water (Carl Roth, France) in a total reaction volume of 30 μl per sample. The thermal cycler conditions for the primary PCRs for bacterial amplification in bulk soil and root samples were 30 cycles of 98°C for 5 s, 78°C for 10 s, 52°C for 20 s, and 72°C for 15 s. The primary PCR conditions for fungal amplification in bulk soil and root samples were 30 cycles of 98°C for 5 s, 78°C for 10 s, 55°C for 20 s, and 72°C for 15 s. PCR products without the addition of microbial DNA (negative control) and mock communities of known fungal or bacterial compositions were added as quality controls. Samples (50 μl; 30 ng of DNA/μl) were sent for tagging and MiSeq Illumina next-generation sequencing using the 2 × 250 paired-end standard operating procedure to GeT-PlaGe INRAe sequencing platform (Toulouse, France).

**TABLE 2:** Sequences of primers and PNA PCR blockers used in this study

Primer	Sequence (5′–3′)
515F_Universal	GTGYCAGCMGCCGCGGTAA
515F_Chloroflexi	GTGCCAGCMGCWGCGGTAA
515F_TM7	GTGCCAGCMGCCGCGGTCA
515F_Nano	GTGGCAGYCGCCRCGGKAA
806R_Universal	GGACTACNVGGGTWTCTAAT
806R_Nano	GGAMTACHGGGGTCTCTAAT
ITS3NGS1	CATCGATGAAGAACGCAG
ITS3NGS2	CAACGATGAAGAACGCAG
ITS3NGS3	CACCGATGAAGAACGCAG
ITS3NGS4	CATCGATGAAGAACGTAG
ITS3NGS5	CATCGATGAAGAACGTGG
ITS3NGS10	CATCGATGAAGAACGCTG
ITS4NGS	TCCTSCGCTTATTGATATGC
pPNA_717-1B4	GGCTCAACCCTGGACAG
mtPNA 717-1B4	GGCAAGTCTTCTTCGGA
ITSspacePNA_717-1B4	CGAGGGCACGTCTGCCTGG

### Sequence processing.

Bacterial and fungal raw sequences were further processed with FROGS (Find Rapidly OTU with Galaxy Solution) (69) implemented on the Galaxy analysis platform (70). Sequences were demultiplexed and dereplicated, the sequence quality was checked, and oligonucleotides, linker, pads, and barcodes were removed from sequences. The sequences then removed from the data set, if they were nonbarcoded, exhibited ambiguous bases or did not match expectations in amplicon size, meaning 50 to 700 nucleotides for fungal sequences and 280 to 500 for bacterial sequences. The remaining sequences were clustered into OTUs based on the iterative Swarm algorithm, and then chimeras and phiX contaminants were removed. OTUs with a minimum number of reads greater than 5 × 10^−5^ percent of total abundance were kept for further analyses for both bacterial and fungal OTUs, as proposed by Escudié et al. (69). The fungal sequences were further processed using the ITSx filter implemented in FROGS in order to discard sequences where an ITS region has not been detected. Bacterial double affiliation was performed by BLAST searching OTUs against SILVA database (71), whereas the UNITE Fungal Database (72) was used for fungal double affiliations. OTUs with a BLAST identity of <85% for bacteria and <80% for fungi were considered chimeras and were removed from the data set. The BLAST identity percentage was considered lower for fungal affiliation in order to keep combined sequences allowed by FROGS. Finally, OTUs corresponding to chloroplasts or mitochondria were removed from the data set. For both fungal and bacterial data, per-sample rarefaction curves were produced to assess sampling completeness, using function *rarecurve*() in the package Vegan v3.5-1 (73) in R (version 3.4.3 [74]). Samples with an insufficient number of sequences according to the rarefaction curves were removed.

Based on these, subsequent analyses of diversity and community structure were performed on data sets where samples had been rarefied with the Phyloseq (75) package to achieve equal read numbers according to the minimum number of total reads in any sample (8,245 reads for fungi and 13,980 reads for bacteria). The microbial community composition and structure in bulk soil and root data were further analyzed by using the Phyloseq package (75).

FUNGuild (76) was used to classify each fungal OTU into an ecological guild. OTUs identified to a guild with a confidence ranking as “highly probable” or “probable” were conserved in our analysis, whereas those ranking as “possible” or with multiple assignations were called “unidentified.” In this study, we focused on the evolution of the distribution of saprotrophs, endophytes, and EcM fungi by averaging their relative abundances (± the standard errors [SE]) between each biological replicate at the same time point. Other fungal types were classified as “unidentified.”

A special procedure was used for AM reads because they were lost in the cleaning process. Therefore, in order to extract reads from AM fungi, the same analysis was repeated with a threshold of minimum abundance of 1 × 10^−6^ percent, and the data generated were only used for AM analysis.

### Statistical analyses.

Statistical analyses and data representations were performed using R software (74; R studio v1.2.5001). Significant differences in the mycorrhization rate between root samples collected over time were detected by checking the normality of the data distribution with Shapiro-Wilk test followed by one-way analysis of variance (ANOVA) and Tukey honestly significant difference (HSD) tests. Differences in fungal and bacterial community structures from bulk soil to roots collected after 2 days (T2) were tested by using the Wilcoxon rank sum test. Differences in fungal and bacterial community structures over time (from T2 to T50) were tested using permutational multivariate analysis of variance (pairwise PERMANOVA) based on binary distances, and differences in structures were visualized using a nonmetric dimensional scaling (NMDS) ordination, using Jaccard (based on presence/absence) and Bray-Curtis (condisidering OTU presence/absence, as well as the relative abundance of OTU) dissimilarity matrices. The Kruskal-Wallis test, followed by a Benjamini-Hochberg correction (false discovery rate correction), and Fisher least significant difference (LSD) *post hoc* tests were used to detect significant differences in the relative abundance of fungal and bacterial phyla, orders, and genera of the bulk soil and across root systems collected at the different time points. This procedure was also used to compare the relative abundance of fungal guilds between root samples collected at the different time points with the difference that Bonferroni correction was applied instead of the Benjamini-Hochberg correction. Variations in fungal and bacterial diversity and richness were tested using the Kruskal-Wallis test, followed by a Bonferroni correction, with Fisher LSD *post hoc* tests. Heatmaps of taxonomic relative abundances were produced using the R package pheatmap (77), and cladograms were built based on Ward’s minimum variance hierarchical clustering. Also, alluvial plots representing the bacterial and fungal relative abundance were produced using the ggalluvial package in R (78). Sparse partial least squares regression (sPLS) methodology was used to look for associations between bacterial and fungal communities (mixOmics package [79]). Correlations between the number of reads of specific microbial taxa were calculated by using a Spearman correlation test.

### Data availability.

Raw data were deposited in the NCBI Sequence Read Archive (SRA) under SRA accession numbers SRR12474095 to SRR12474100 for the 16S data and SRR12474163 to SRR12474204 for the ITS data (project PRJNA657694).

## Supplementary Material

Supplemental file 1

Supplemental file 2
