# Supplementary material for: Colonization of Naive Roots from Populus tremula × alba Involves Successive Waves of Fungi and Bacteria with Different Trophic Abilities
Source: Appl Environ Microbiol. 2021 Feb 26;87(6):e02541-20. doi: 10.1128/AEM.02541-20 (PMC8105020; doi:10.1128/AEM.02541-20)
Supplement: Supplemental file 1 [file AEM.02541-20-s0001.pdf]

Supplemental Figure legends

Figure S1 - Experimental design and approach used in this study.

Figure S2 - Confocal microscopy image of axenic *Populus tremula* x *alba* adventive root at the beginning of the experiment (T0). Plant cell walls were stained with propidium iodide and appear in red. Ar, Adventive Root; Vc, Vascular cylinder

Figure S3 - Melanised septed fungal hyphae colonizing poplar roots from 4 to 30 days of culture. Optic and confocal microscopy images of poplar roots colonized by melanised septed fungal hyphae. A. External colonization of roots by melanised septed fungal hyphae after 4 days of culture. B. Extracellular melanised septed hyphae surrounding an adventive root at 10 days of culture. C. Extracellular melanised septed hyphae after 21 days of culture. Arrow indicates the septa. D. Intracellular melanised septed hyphae propagating in the apoplastic compartment after 21 days of culture. Arrow indicates the ~~DSE~~ septa. E. Extracellular melanised septed hyphae surrounding an EcM forming on a lateral root after 30 days of culture. Arrow indicated the hyphae. E, F. Overlay of image and the green track in order to visualize both melanized and non-melanized fungal structures. Non-melanized fungal structures appear in green through WGA-Alexa Fluor 488 staining. Ar, Adventive root; Hp, Hyphae; Lr; Lateral root; Rh, Root hair.

Figure S4 - Intermediate stage of the fungal colonisation dynamic. Confocal microscopy images of poplar roots colonised by fungi after 7 to 15 days of culture. A. EcM formation on a lateral root after 15 days of culture. B. Hartig net formation on EcM after 15 days of culture. C. Co-existing and abundant fungal morphologies within the same root region after 15 days of growth. Orange arrow indicates the « arbuscular-like » and white arrow indicates the « hand glove-like » fungal structures. Fungal structures appear in green

through WGA-Alexa Fluor 488 staining while root cell-walls were stained with propidium iodide and appear in red. Ar, Adventive root; Ap, Apex; Hn, Hartig net; Hp, Hyphae; Lr; Lateral root, Mt, Mantle; Rc, Root cell; Vc, Vascular cylinder

Supplemental Table legends

Table S1 – Relative abundance of bacterial communities at each taxonomic rank detected in the bulk soil and/or roots samples collected from T2 to T50. Each given value is the average value of 3, 4 or 5 replicates ± SE. The second column denote significant differences between bulk soil samples and root samples collected from T2 and the third column denote significant difference in relative abundance of fungal communities between each sampling time from T2 to T50. Different letters denote significant difference between each sampling time (Kruskal-Wallis, Benjamini and Hochberg correction, Fisher's LSD post-hoc test, *P*.adj<0.05).

Table S2 – Relative abundance of fungal communities at each taxonomic rank detected in the bulk soil and/or roots samples collected from T2 to T50. Each given value is the average value of 3, 4 or 5 replicates ± SE. The second column denote significant differences between bulk soil samples and root samples collected from T2 and the third column denote significant difference in relative abundance of fungal communities between each sampling time from T2 to T50. Different letters denote significant difference between each sampling time (Kruskal-Wallis, Benjamini and Hochberg correction, Fisher's LSD post-hoc test, *P*.adj<0.05).

Table S3 – Distribution of the relative abundance of fungal guilds detected in the bulk soil samples and in the roots samples collected from T2 to T50. Each given value is the average value of 3, 4 or 5 replicates ± SE. The second column denote significant differences between bulk soil samples and root

samples collected from T2 and the third column denote significant difference in relative abundance of fungal communities between each sampling time from T2 to T50 (Kruskal-Wallis, Bonferroni correction, Fisher's LSD post-hoc test, *P*.adj<0.05).

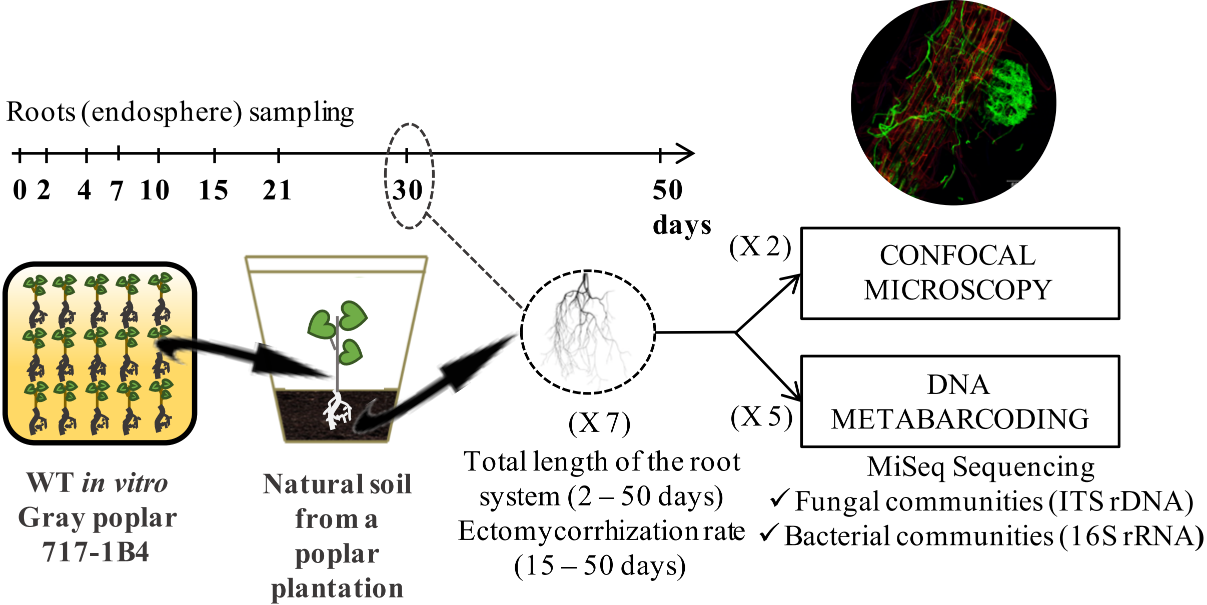

Roots (endosphere) sampling
0 2 4 7 10 15 21 30 50
days
(X 2)
CONFOCAL MICROSCOPY
(X 5)
DNA METABARCODING
(X 7)
WT in vitro Gray poplar 717-1B4
Natural soil from a poplar plantation
Total length of the root system (2 – 50 days)
Ectomycorrhization rate (15 – 50 days)
MiSeq Sequencing
✓ Fungal communities (ITS rDNA)
✓ Bacterial communities (16S rRNA)

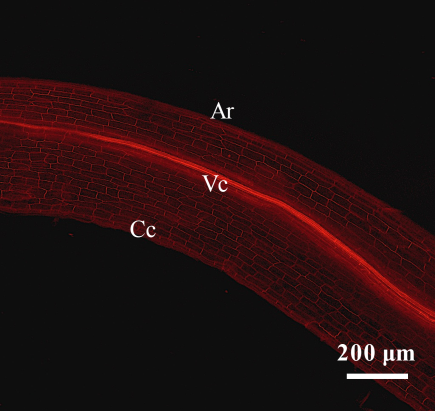
Ar
Vc
Cc
200 µm

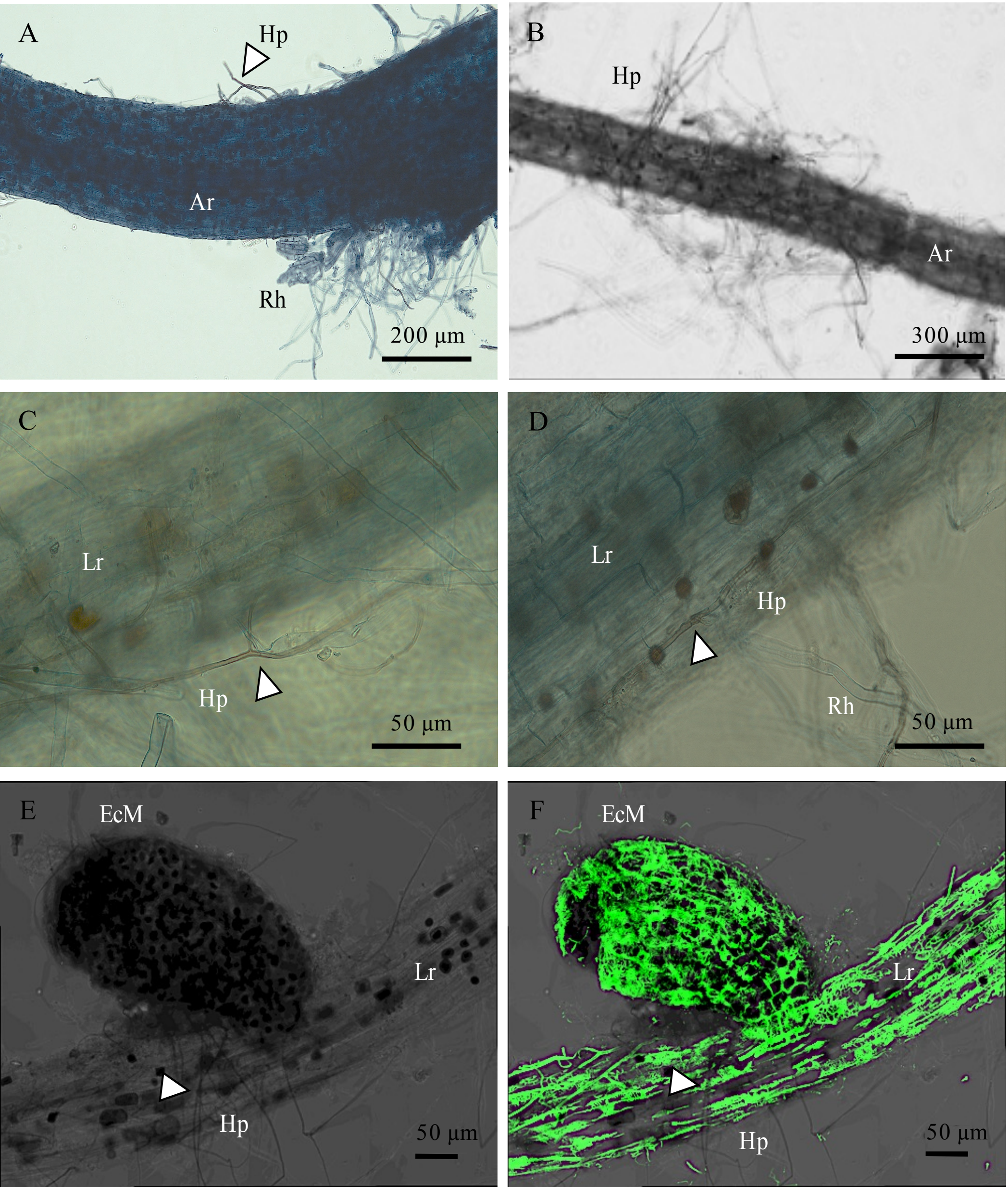

A
Hp
Ar
Rh
200 µm
B
Hp
Ar
300 µm
C
Lr
Hp
50 µm
D
Lr
Hp
Rh
50 µm
E
EcM
Lr
Hp
50 µm
F
EcM
Lr
Hp
50 µm

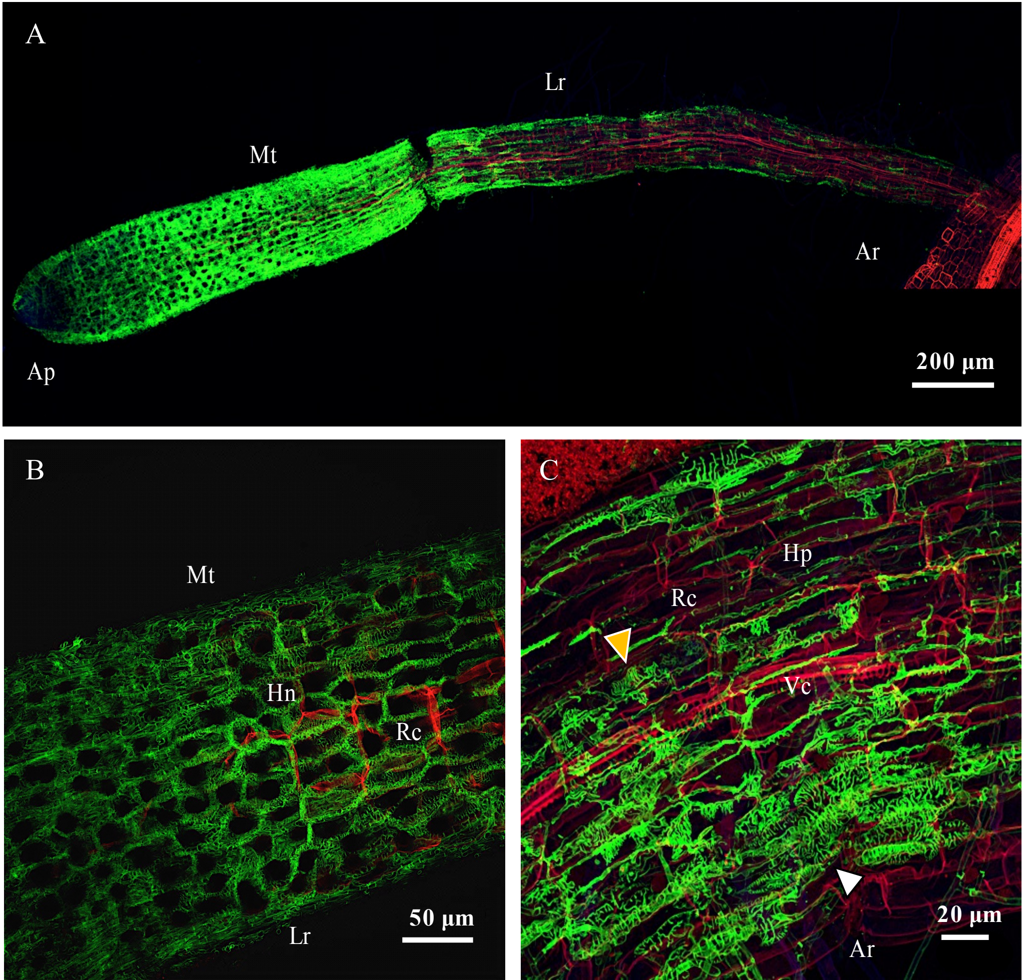
A
Lr
Mt
Ar
Ap
200 µm
B
Mt
Hn
Rc
50 µm
Lr
C
Hp
Rc
Vc
20 µm
Ar
